# Role of NO/cGMP signalling in VEGF-mediated angiogenesis

**DOI:** 10.1186/1471-2210-11-S1-P35

**Published:** 2011-08-01

**Authors:** Ronald Jäger, Dieter Groneberg, Andreas Friebe

**Affiliations:** 1 Physiologisches Institut, Universität Würzburg

## Background

NO-sensitive guanylyl cyclase (NO-GC) is the most important effector protein for nitric oxide (NO) and is involved in various physiological processes. Binding of NO stimulates NO-GC up to 200-fold and thereby increases cGMP synthesis. NO is generated by different NO synthases (NOS). In the vasculature, endothelial NOS (eNOS) is involved in the regulation of blood pressure by relaxation of vascular smooth muscle. eNOS is known to be stimulated by shear stress. In addition, NO synthesis is stimulated by vascular endothelial growth factor (VEGF) which acts as one of the most potent stimulators of angiogenesis.

Until now the exact signalling pathway of VEGF-induced NO has not been elucidated. S-nitrosylation of protein thiols as well as stimulation of NO-GC with subsequent cGMP increases may underlie the angiogenic effect of VEGF.

## Method

In order to investigate the involvement of the NO/cGMP cascade in VEGF-mediated angiogenesis we generated an endothelial-specific NO-GC knock-out mouse (EC-GCKO) using the Tie2-Cre line.

## Results

Preliminary macroscopic analysis revealed that EC-GCKO mice are indistinguishable from WT animals and do not show any of the gross phenotypes of the total GCKO mice. Several in vitro and in vivo techniques are being used such as primary endothelial cell culture, aortic ring assay and matrigel plug analysis.

